# THIRSTY FOR FRUCTOSE: Arginine Vasopressin, Fructose, and the Pathogenesis of Metabolic and Renal Disease

**DOI:** 10.3389/fcvm.2022.883365

**Published:** 2022-05-17

**Authors:** Jeffrey Student, James Sowers, Warren Lockette

**Affiliations:** ^1^Drexel University College of Medicine, Philadelphia, PA, United States; ^2^Division of Endocrinology, University of Missouri School of Medicine, Columbia, MO, United States; ^3^Division of Endocrinology, Wayne State University School of Medicine, Detroit, MI, United States

**Keywords:** fructose, vasopressin (ADH), Mesoamerican nephropathy, metabolic syndrome, high fructose corn syrup (HFCS)

## Abstract

We review the pathways by which arginine vasopressin (AVP) and hydration influence the sequelae of the metabolic syndrome induced by high fructose consumption. AVP and inadequate hydration have been shown to worsen the severity of two phenotypes associated with metabolic syndrome induced by high fructose intake–enhanced lipogenesis and insulin resistance. These findings have implications for those who frequently consume sweeteners such as high fructose corn syrup (HFCS). Patients with metabolic syndrome are at higher risk for microalbuminuria and/or chronic kidney disease; however, it is difficult to discriminate the detrimental renal effects of the metabolic syndrome from those of hypertension, impaired glucose metabolism, and obesity. It is not surprising the prevalence of chronic renal insufficiency is growing hand in hand with obesity, insulin resistance, and metabolic syndrome in those who consume large amounts of fructose. Higher AVP levels and low hydration status worsen the renal insufficiency found in patients with metabolic syndrome. This inter-relationship has public health consequences, especially among underserved populations who perform physical labor in environments that place them at risk for dehydration. *MesoAmerican endemic nephropathy* is a type of chronic kidney disease highly prevalent in hot ambient climates from southwest Mexico through Latin America. There is growing evidence that this public health crisis is being spurred by greater fructose consumption in the face of dehydration and increased dehydration-dependent vasopressin secretion. Work is needed at unraveling the mechanism(s) by which fructose consumption and increased AVP levels can worsen the renal disease associated with components of the metabolic syndrome.

## Introduction

**Figure 1:**
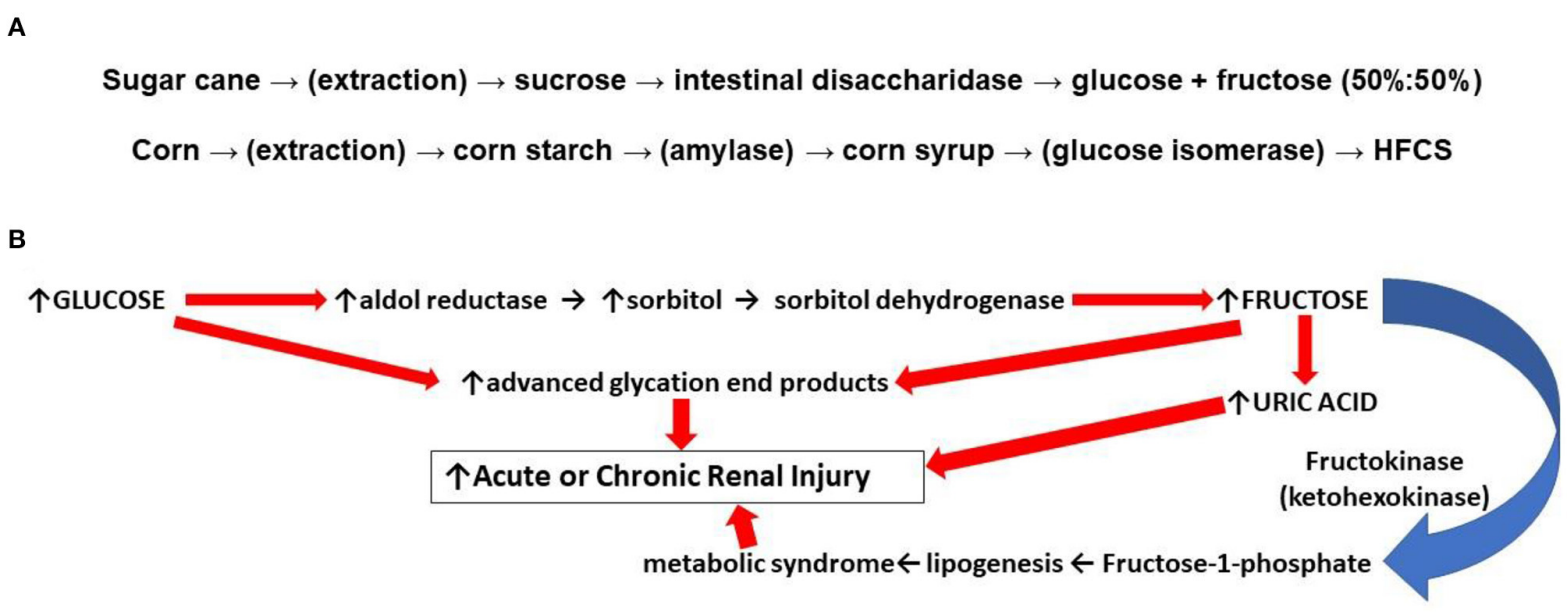
**(A)** (top): Differences in food sweeteners from sugar and corn. Sucrose is extracted from sugar cane or beets. Ingested sucrose from processed foods is digested in the small intestines by disaccharidase into equal parts glucose and fructose (50%:50%). Absorbed fructose is almost completely taken up by GLUT-1 and GLUT-5 transporters in the liver which accounts for low levels of plasma fructose. Corn starch is a polysaccharide that is enzymatically hydrolyzed to monosaccharides which in the presence of isomerase increases the relative content of fructose yielding a high fructose corn syrup (HFCS). **(B)** (bottom): Some mechanisms by which fructose can result in renal injury. The endogenous conversion of glucose to fructose through the polyol pathway contributes to formation of uric acid, which promotes mitochondrial oxidative stress and inflammation. Endogenous fructose generation also promotes lipogenesis which contributes to the development of the metabolic syndrome. Glucose and fructose can both react directly with cellular lipids and proteins; oxidized LDL and collagen cross linked by these reactions can induce vascular stiffness, stimulate inflammation, and promote atherogenesis (4).

The incidence of obesity and metabolic syndrome related renal disorders has increased in recent years. A recent cross-sectional analysis from 2011 to 2016, found the prevalence of metabolic syndrome in the United States to be 34.7% of adults (1). Meanwhile, from 1986 to 2000, the biopsy incidence of obesity-related glomerulopathy increased 10-fold, from 0.2 to 2% (2). It is believed that the rising prevalence of obesity comes from excess consumption of sugars, and especially fructose (2, 3). It is important to understand the causes of increased fructose consumption in men and women. Fructose, “fruit sugar,” is a hexose monosaccharide with the same molecular formula as glucose (C_6_H_12_O_6_). High fructose *corn* syrup (HFCS) is a manufactured sweetener that contains a mixture of the monosaccharides, glucose and fructose, in varying ratios (e.g., 45%:55%). In “sweetness,” HFCS is similar to *cane* sugar (sucrose), a disaccharide first hydrolyzed in the small intestines before systemic absorption; however sucrose digestion yields glucose and fructose in equal parts (i.e., 50%:50%, Figure 1A) (5). The average fructose content of popular beverages sweetened with HFCS is 59% with several major brands approaching 65% (6). Relative to sucrose extracted from cane sugar, high fructose, corn-based syrup is less expensive, and the low cost explains its widespread use. Although excess intake of both HFCS and sucrose from cane sugar is linked to the metabolic syndrome, some have argued that the incrementally greater percentage of fructose in HFCS compared to sucrose puts those at greater risk of metabolic disease when consuming more HFCS relative to sucrose. In either case, an understanding of the role fructose plays in the pathogenesis of metabolic syndrome is needed.

Several potential mechanisms by which the increased consumption and metabolism of fructose can worsen the phenotypic expression of the metabolic syndrome relative to glucose have been proposed. First, advanced glycation end products (“AGE's”) are irreversible molecular adducts, damaging to cells, that are formed from the nonenzymatic reaction between reducing sugars such as fructose and glucose with proteins or lipids. Whereas, glucose contains an aldehyde, fructose has a ketone group which makes it even more reactive with amines found in proteins (the non-enzymatic “Maillard reaction”) (4). Second, when fructose is administered exogenously, the sugar is taken up by the liver and sequentially metabolized by fructokinase and aldolase with the rapid consumption of adenosine triphosphate (ATP) (7). A transient depletion of ATP and intracellular phosphate shunts adenosine and inosine to purine degradation pathways and the synthesis of uric acid; the increased production of uric acid is accompanied by mitochondrial oxidative stress and inflammation in the liver (8, 9). Furthermore, in multiple tissues, uric acid can promote the metabolism of glucose through the polyol pathway where, in the face of high glucose concentrations, glucose is reduced to sorbitol with its subsequent oxidation to fructose leading to further hepatic steatosis or renal damage (Figure 1B) (10, 11). Uric acid can also inhibit insulin signaling and induce insulin resistance (12).

## Arginine Vasopressin, Fructose, and the Metabolic Syndrome

**Figure 2:**
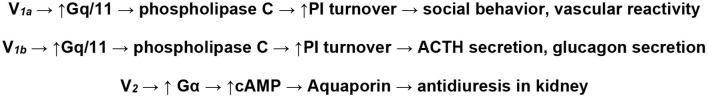
There are three subtypes of the G-protein linked vasopressin receptors. V_1*a*_ receptors are found on vascular smooth muscle and platelets and induce vasoconstriction through increased phosphatidyl inositol (PI) metabolism; V_2_ receptors are found primarily in on principal cells of the kidney collecting ducts and mediate water reabsorption through aquaporins stimulated by AVP in the systemic circulation; V_1*b*_ receptors are found primarily in cells of the pituitary, white adipose tissue, and the pancreas. AVP secreted from parvocellular neurons and flowing through the hypophyseal portal circulation stimulates pituitary adrenocorticotropic hormone (ACTH) secretion through interaction with V*1b* receptors in pituitary corticotrophs by potentiating the stimulatory effects of corticotrophin-releasing hormone on ACTH.

Arginine vasopressin (AVP) is now being implicated in the development of metabolic syndrome. AVP is produced by two groups of cells—neurons of the hypothalamic paraventricular nuclei (PVN) and the supraoptic nuclei (SON). These neurons are divided into either the magnocellular system secreting AVP into the *peripheral circulation* from axon terminals within the pituitary, and also, the parvocellular system with axons projecting to the median eminence of the hypothalamus where AVP is secreted into the *pituitary portal circulation* (13, 14). Through the systemic circulation, or alternatively, through the pituitary portal system, AVP activates three distinct vasopressin receptors, V*1a*, V*1b*, and V*2*, encoded by three distinct genes in humans, on chromosomes 12q14.2, 1q32.1, and Xq28, respectively, with variation in the mechanisms of post-receptor signal transduction (Figure 2) (15–17).

There are several pathways by which increases in circulating AVP can contribute to the development of the metabolic syndrome. Indeed, over 10 years ago, it was recognized that AVP levels significantly correlated with several markers of the metabolic syndrome, i.e., body mass index, fasting plasma glucose and insulin concentrations, insulin resistance, and triglyceride levels. Vasopressin has been shown to increase stress-induced ACTH secretion in a V_1b_ dependent manner, and repetitive stress is associated with the development of the metabolic syndrome (18, 19). Subsequently, it has been shown that copeptin, the stable carboxy-terminal portion of pro-arginine vasopressin secreted in equimolar amounts with AVP, is independently associated with hyperinsulinemia, the development of diabetes mellitus, and the metabolic syndrome (20–22). Because hydration levels mediate central AVP release and plasma AVP concentrations, it is not surprising that hydration levels have also been shown convincingly to correlate with the presence of the metabolic syndrome (23–33).

AVP has been shown to stimulate of hepatic gluconeogenesis and glycogenolysis through the activation of hepatic V_1*a*_ receptors, and also contributes to the secretion of glucagon or insulin through the activation of pancreatic V_1*b*_ receptors. These findings are consistent with the observation that sustained AVP infusion in laboratory rodents induced both a time and dose-dependent increase in fasting blood glucose concentrations, whereas lowering endogenous AVP by increasing water intake had no effect. In the same study, the effect of AVP on fasting blood glucose was attenuated by cotreatment with a V_1*a*_ receptor antagonist. This may help explain the reported observations of elevated plasma AVP (copeptin) levels in patients with diabetes (34). It is likely that these findings, that AVP increases glycemia through the activation of V_1*a*_ receptors, are mediated by the activation of *hepatic* V_1*a*_ receptors and the stimulation of hepatic gluconeogenic processes. However, further study is needed to confirm this is a purely hepatic process. AVP also acts synergistically with central V_1*b*_ receptors to not only promote ACTH secretion, as noted above, but also catecholamine secretion from the adrenal medulla In one clinical study, 31 healthy adults with high copeptin levels, low 24 h urine volumes, and high urine osmolality were recruited to consume an additional 1.5 L of water daily for 6 weeks. Although the intervention did not significantly affect fasting ACTH, cortisol, fasting glucose, insulin, or glucagon concentrations, changes in copeptin levels were highly associated with changes in ACTH levels. These findings also make tenable that central AVP concentrations in the pituitary portal circulation can contribute to ACTH-mediated stress responses and the development of the metabolic syndrome (35). In summary, there appears to be both metabolic (mediated by V_1*a*_ receptor-induced gluconeogenesis) and central (mediated by activation of V_1*b*_ receptors and the stress response) mechanisms by which AVP contributes to hyperglycemia and the metabolic syndrome.

As noted, the V_1*b*_ receptor plays a critical role in regulating hypothalamic-pituitary-adrenal axis activity by contributing to pituitary ACTH secretion and corticosterone release from the adrenal gland levels under basal and stress conditions (18). Indeed, the normal increase in circulating ACTH levels in response to AVP is impaired in V_1*b*_ receptor knockout mice, although corticotropin-releasing hormone (CRH)-stimulated ACTH release is not impaired. Vasopressin levels and V_1*b*_ receptor expression were elevated in rodents with exposure to chronic stress, and the measured increase in ACTH after stress (e.g., after a forced swim test) has been found to be significantly suppressed in V_1*b*_ receptor null mice (18, 36). Immediately after a stressful event, there is a CRH-mediated suppression of food intake, followed by a CRH/ACTH-dependent, glucocorticoid mediated, stimulation of hunger and eating behavior (37). Accordingly, chronic stress, and the resultant chronic activation of the hypothalamic-pituitary adrenal axis, induces weight gain in rodents and humans where it is known as “emotional eating” or “stress-induced eating” (38–41). In addition to the hyperglycemic effects of AVP described above, it is likely that AVP produces *behavioral* changes via V_1*b*_ potentiation of the CRF-ACTH-glucocorticoid axis.

Teleologically, the secretion of AVP has evolved as a mechanism to ensure appropriate hydration status and fluid balance. Because a significant amount of water is endogenously provided through the catabolism of fat, specifically through fatty acid oxidation, it is intriguing to speculate that AVP would play a role at increasing fat storage (42). Indeed, it is one reason posited to explain the evolution of the camel's hump, which is a fat storage depot (43). It is noteworthy that fructose stimulates the secretion of AVP to a greater degree than glucose. In hypothalamic explants, fructose directly stimulates AVP secretion (44). When rodents are slowly infused with a hypertonic 20% solution of either glucose or fructose, AVP levels only rise in response to the administration of fructose. This is likely due to the facilitated uptake of glucose, relative to impermeant fructose, into AVP-releasing neurons sensing osmolality (45).

It has also been shown that the effect of AVP on inducing hepatic lipogenesis and the metabolic syndrome is dependent upon the V_1*b*_ receptor. In a study utilizing vasopressin receptor knockout mice being fed high fructose diets, V_1*b*_ receptor null mice were found to have suppressed markers of the metabolic syndrome phenotype, including fat mass, hepatic steatosis, serum triglycerides, transaminases, insulin, and leptin. Interestingly, these same markers were found to be elevated in V_1a_ receptor deficient mice, suggesting that while V_1*b*_ receptors promote lipogenesis and the development of the metabolic syndrome, activation of V_1*a*_ receptors may play a protective, counterregulatory role (42). These findings from rodents were corroborated by the study of overweight and obese subjects who consumed glucose- or fructose-sweetened beverages providing 25% of energy requirements for 10 weeks. Although both groups exhibited similar weight gain during the intervention, visceral adipose volume was significantly increased only in subjects consuming fructose, and hepatic de novo lipogenesis was increased specifically during fructose consumption (46). Fructose is a secretagogue for AVP, and AVP promotes lipogenesis, hyperinsulinemia, and many of the pathophysiological intermediaries associated with the metabolic syndrome.

Very little orally ingested fructose hits the cerebral circulation, therefore the effect of endogenous fructose production on AVP secretion should be considered. Exogenous fructose is preferentially taken up by glucose transporters (especially GLUT5) and metabolized in the liver while glucose, transported by GLUT1 and GLUT3, is metabolized primarily in the brain (47). Accordingly, plasma fructose concentrations are typically 0.01% of plasma glucose levels. Meanwhile, glucose concentrations in cerebrospinal fluid (CSF) are roughly 60% of its plasma concentrations whereas CSF levels of fructose are twenty times greater than those levels found in the blood. It is suggested from these observations that because fructose is so rapidly metabolized by the liver, endogenous fructose generation by the brain is physiologically significant (48). Indeed, both the catabolism of fructose by fructokinase and synthesis of fructose by the polyol pathway have been demonstrated in the brain (49). Clearly, endogenous fructose production in the brain can contribute to any AVP-dependent pathway that promotes the metabolic syndrome phenotype despite having low plasma concentrations of fructose.

## Arginine Vasopressin, Fructose, and Renal Insufficiency

*The potential potentiation of the metabolic syndrome from chronically increased levels of AVP, albeit at low levels, and increased fructose consumption has significant public health implications* (50). This is particularly true in hot, arid lands, such as Mesoamerica where high ambient temperatures and physical exertion, and accordingly high levels of AVP, are associated with the growing prevalence of the metabolic syndrome and the development of chronic kidney disease (51, 52). Indeed, *Mesoamerican endemic nephropathy* is a type of chronic kidney disease of unknown origin that is highly prevalent among manual laborers from southwest Mexico through Latin America (53, 54). In fact, study of manual laborers in Nicaragua revealed 14% of all men had an estimated glomerular filtration rate (eGFR) of <60 mL/min/1.73 m^2^ (55). Similar findings were yielded in a study of El Salvadorian coastal communities, where 18% of men had an eGFR <60 mL/min/1.73 m^2^ (56). As a chronic kidney disease of nontraditional origin (CKDnt), the incidence and public health burden of Mesoamerican endemic nephropathy cannot be attributed to classical risk factors such as hypertension or diabetes. The exact cause of CKDnt has yet to be determined, and recurrent heat stress/dehydration and agrochemicals remain among the most widely discussed risk factors (57). The Pan American Health Organization (PANAHO) has reported “the death toll of the epidemic of CKDnt in Mesoamerica runs into the tens of thousands, affecting mostly young men.” Because this deteriorating renal disease is observed mostly in plantation workers exposed to physically stressful work environments and high ambient temperatures, PANAHO has further gone on to recommend a “water-shade-rest” intervention to farm workers (58).

The increased consumption of sweetened beverages is associated with urbanization and economic growth. As such, more intensified policy efforts are needed to ensure that hydration occurs with water, not through sweetened beverages that increase the intake of sugar and, subsequently, increase the global burden of obesity and chronic diseases. There is growing evidence that hydration with beverages sweetened with high fructose corn syrup potentiates the renal insufficiency found in those who must toil in high ambient temperature climates. However, as any traveler to an underdeveloped country has observed, the advertising for sugar sweetened beverages is ubiquitous, equaled only by the fervent pitches for various cell phone carriers. In the United States, it is estimated that the average American consumes roughly 54.7 g of fructose per day and nearly 30 kg of carbohydrate each year from added sweetners (59, 60). In Latin America, average individual sugar intake is estimated at 99.4 g/day, with an average of 65.5 g/day coming from added sugar (61).

In a study with laboratory rodents pair fed a diet containing 60% fructose, 60% dextrose, or standard rat chow, the fructose fed rats had worse outcomes in a reduced renal mass model of chronic kidney disease. Proteinuria was increased and creatinine clearance was diminished in the rodents fed fructose when compared to glucose- and control-fed rodents. Glomerulosclerosis, tubular atrophy, tubular dilatation, and inflammation were all greater in the fructose fed rats (62). The toxic effect of fructose is not limited to injured kidneys as dietary fructose has been shown to produce a similarly pathologic effect on *normal* rodent kidneys (63). Endogenous fructose generation via the polyol pathway may be particularly pathologic because, in addition to the production of fructose, this pathway may also increase the relative abundance of sorbitol, which plays a role in directly inducing renal injury (64). In a study of the potential role of endogenous fructose production, as opposed to dietary fructose, wild-type mice with streptozotocin-induced diabetes were shown to develop proteinuria, reduced glomerular filtration rate, and renal glomerular and proximal tubular injury. However, unlike control mice, rodents genetically deficient in fructokinase demonstrated significantly less proteinuria, renal dysfunction, renal injury, and inflammation. These studies further support a role for fructokinase and its metabolites as a novel mediator of diabetic nephropathy (65).

Fructose can directly stimulate AVP release or induce the secretion of AVP by the hyperosmolarity generated by increasing sorbitol concentrations via the polyol pathway. Conversely, genetic ablation of fructokinase diminishes AVP secretion induced by dehydration (45). Given this intimate relationship between AVP and fructose, it is necessary to understand what effect fructose has on the physiologic responses to dehydration in men and women if we are to understand the endemic nephropathy of Mesoamerica. In one study, mice were exposed to repetitive heat stress for 5 weeks. Compared to control animals given water, there was a progressive worsening of renal inflammation and fibrosis in mice given rehydration with 10% fructose in water. It was suggested that following heat-induced renal injury, oral fructose ingestion may accelerate kidney damage (66). Another study examining heat-induced renal injury found that compared to water, rehydration with fructose following heat stress resulted in significant decrements in renal blood flow (measured via Doppler ultrasound) and creatinine clearance along with significantly elevated renal vascular resistance. Interestingly, co-administration of conivaptan, a nonselective vasopressin receptor antagonist, prevented these changes, suggesting fructose mediated renal injury following heat stress occurs in an AVP-dependent fashion (67).

Many of these findings have been supported by additional study. Fluid restriction followed by water hydration mildly increased urine osmolality and induced a 15% decrement in creatinine clearance while increasing two markers of tubular damage, urinary N-Acetyl-β-D-glucosaminidase and kidney injury molecule-1. These changes were accompanied by overexpression of V_1*a*_ and V_2_ renal receptors, the polyol fructokinase pathway, and increased renal oxidative stress with reduced expression of antioxidant enzymes. Hydration with an unspecified 11% glucose/fructose sweetened beverage significantly amplified those alterations, and it is suggested that current habits of re-hydration with sweetened beverages could be a risk factor in developing kidney damage (68).

The findings in rodents regarding fructose and renal injury have been recapitulated in men and women. In one study of 12 healthy adults, subjects drank 2 liters of a fructose containing soft drink or water during 4 h of exercise. Serum creatinine increased ≥0.30 mg/dl post exercise in 75% of participants in the soft drink trial compared with 8% in water trial, which is consistent with the Kidney Disease Improving Global Outcomes definition of acute kidney injury (69). Changes in serum uric acid from pre-exercise were greater in the soft drink trial than the water trial at post-exercise, and there were greater increases from pre-exercise to post-exercise in serum copeptin in the soft drink trial. It was suggested that consuming a fructose beverage during and following exercise in the heat induces kidney injury through a vasopressin-mediated mechanism, which was consistent with the findings observed in rodents (70). Subsequently,13 healthy subjects who consumed 500 mL of a HFCS-sweetened soft drink had augmented kidney vasoconstriction (measured by Doppler ultrasound) to sympathetic stimulation induced with the cold pressor test when compared to subjects who consumed water. During a separate trial, venous blood samples were obtained in 12 healthy adults before and 30 min after consumption of 500 mL water with artificial sweetener, sucrose, or high fructose corn syrup. Increases in serum uric acid were greater in those who consumed high fructose corn syrup compared to those who consumed artificial sweetener or sucrose.

In summary, it is suggested that high fructose corn syrup acutely increases vascular resistance in the kidneys with simultaneous elevations in circulating uric acid and vasopressin (71). There are consistent suggestions that hypohydration and rehydration with fructose-based sweetened beverages facilitate the secretion of AVP and predispose humans to metabolic syndrome and the development of renal insufficiency. Despite such consistent suggestions, our understanding of this relationship and the mechanisms by which fructose and vasopressin could induce metabolic and renal disease remain incomplete. Further study utilizing selective and nonselective vasopressin agonists and antagonists could prove useful in characterizing the physiological changes produced by AVP, establishing the precise mechanisms by which AVP verses fructose contributes to renal disease, and identifying the utility of AVP receptor blockade to combat diseases such as Mesoamerican endemic nephropathy. Fructose could be a key mediator in this cascade and the mechanisms by which it exerts its toxic effects warrants further exploration. It is worth noting that exogenous AVP is frequently administered, in the absence of fructose, as a means of pressor support in critically ill patients and has been found to protect against renal injury in this setting (72). Finally, further epidemiological study of fructose consumption and renal insufficiency, particularly in Mesoamerica, could help reduce the impact of CKDnt and obesity related kidney disease. Regardless, as evidenced by this review, there is ample evidence to suggest fructose and AVP contribute to the development of metabolic and renal disease. We should not be thirsty for fructose, but instead, thirsty for water.

## Author Contributions

All authors listed have made a substantial, direct, and intellectual contribution to the work and approved it for publication.

## Funding

This work was supported, in part, by the Department of Defense and Henry M. Jackson Foundation for the Advancement of Military Medicine, HU0001-18-2-0016, HJF Award, 309833-1.00-65546.

## Conflict of Interest

The authors declare that the research was conducted in the absence of any commercial or financial relationships that could be construed as a potential conflict of interest.

## Publisher's Note

All claims expressed in this article are solely those of the authors and do not necessarily represent those of their affiliated organizations, or those of the publisher, the editors and the reviewers. Any product that may be evaluated in this article, or claim that may be made by its manufacturer, is not guaranteed or endorsed by the publisher.
